# Continuing Professional Development through the lens of complexity science: Becoming agents of change in the healthcare system

**DOI:** 10.15694/mep.2020.000186.1

**Published:** 2020-08-31

**Authors:** Natalie Ong, Janet Long, James Woodruff

**Affiliations:** 1 Children's Hospital at Westmead Clinical School; 2 Australian Institute of Health Innovation; 3 Pritzker School of Medicine

**Keywords:** continuing professional development, in service training, complex adaptive systems, complexity science, staff education, quality improvement, intellectual disability, developmental disability, children

## Abstract

This article was migrated. The article was not marked as recommended.

Healthcare improvement initiatives have not led to expected changes in patient population outcomes over the last decade. This can, in part, be explained by overly reductionist approaches in medical practice and education. This article describes some insights and experiences using a Complex Adaptive Systems approach in the conceptualisation of a continuing professional education program and reflections on elements that led to enhanced practice behaviours and system improvements.

## Continuing Professional Development through the lens of complexity science: Becoming agents of change in the healthcare system

Through the decades, progress in health care quality and performance has been known to be slow. In an article in the BMJ and The Health Foundation Quality Improvement series ( 2018), Braithwaite stated that only “50-60% of care is delivered using level 1 evidence or clinical guidelines; a third of medicine is waste and the rate of adverse events for the last 25 years have remained static at about 10%” ( Braithwaite, 2018). The paper calls for alternate ways of looking at the healthcare system in order to devise new solutions for healthcare challenges ( Braithwaite, 2018). There are many hypotheses as to why healthcare quality has not improved as expected. One hypothesis attributes slow progress to pervasive use of overly reductionist approaches in medical education and clinical practice. Woodruff suggests that we cannot solve complex problems using a reductionist paradigm because complexity exists within all layers of the healthcare system and affects the efforts of clinicians and policy makers to improve and reconcile individuals’ healthcare experience, population outcomes and healthcare costs ( Woodruff, 2019). To better account for the complex nature of medicine, such systems should instead be viewed through a “complex adaptive systems framework” (CAS). Such a framework better anticipates the dynamic interactions between the individual’s biopsychosocial systems and complex layers of the health system ( Kuziemsky, 2016).

Batalden and Davidoff (2007) propose a model that describes the complex relationship between high quality professional development and quality improvement programming characterized by involvement of patients, families, payers, planners, researchers and educators towards improved patient outcomes. They also propose that ‘change’ is dynamic and needs to be an intrinsic part of the healthcare system ( Batalden and Davidoff, 2007). For this to occur, continuing professional development (CPD) activities should include or work synergistically with continuous quality improvement (QI) initiatives characterized by collaborative teams pursuing iterative problem solving. These CPD and QI activities would align with adult learning theory and “Plan Do Study Act” cycles as parallel and complementary processes delivered across time. The idea of introducing new knowledge that builds on current understanding of a topic is then consolidated through the application in practice. This is reviewed and reflected upon over time and offers opportunities for continuous improvements. These insights suggest that clinical educators should review their training program design through a complex adaptive systems lens to better understand these complex interactions between CPD/ QI activities, staff responses and the effects on the health system. Doing so may provide a more comprehensive understanding of how their educational and training activities may lead to more effective behaviour change and systems improvement.

Woodruff proposes a model for applying CAS concepts to medicine. The model outlines four system elements required for self-organising systems or resilient medical organisations tasked with complex problem solving. These include “intrinsic characteristics” that contribute to the mission (expertise, knowledge), an “attractor” (shared values, professionalism), “adaptive capacity” (judgment, responsiveness to the situational context), and “the absence of excessive central control” (professional autonomy, space to respond autonomously to local phenomena). Collectively these elements necessarily result in team oriented continuous quality improvement and endow the system with a capacity to provide ethical, coordinated and personalized care. The absence of any one element, compromises that capacity.

In my experience facilitating in-service training for health staff, I have found that influencing intrinsic motivation as well as increasing one’s technical expertise (“intrinsic characteristics”) work alongside each other through reflective practices and case-based discussions. Through the sharing of experiences and peer norming, this promotes a sense of a common purpose (“attractor”) which then leads to an increase in the capacity to adapt (“adaptive capacity”) ( Woodruff, 2019). We also involved a mix of clinical and management staff in process mapping discussions around staff and patient feedback. This fostered openness, ownership and autonomy (“absence of excessive central control”) as staff were supported to speak up about areas that need improvement or attention to skills development. Using this approach, I have found that learners have an increased desire to learn more, take action and propose unexpected projects and QI initiatives. These activities done over time have set up “feedback loops” where efforts that resulted in success encouraged further efforts. This then led to the emergence of more open and “just” cultures, local champions and early adopters, innovative localised solutions and system improvements. These behaviours if sustained could potentially spread to other individuals or departments, inspiring the same approach and to adopt the habit of lifelong learning and contribute to continuous quality improvement and the development of a dynamic learning organisation.

CPD providers are agents of change for our healthcare system. While their efforts have traditionally focused on enhancing professional competency, they are well positioned to go even further, explicitly empowering learners to enhance patient care and health outcomes. Such efforts will require integration of CPD with core concepts from quality improvement such as systems thinking and complexity science ( Sargeant, Wong and Campbell, 2018). CPD designed with concepts such as CAS in mind has the potential to further transform our healthcare workforce into flexible and adaptable teams committed to a culture of continuous improvement of healthcare for all.

## Take Home Messages

• Progress in health care quality and performance has been slow partly due to the overly reductionist approaches to medical education and clinical practice
• New ways of thinking about the health care system is required to devise new approaches to improvements in health care
• Complexity exists in all layers of the health care system from the biopsychosocial interactions at the level of the individual to the interactions of complex networks within the health system
• CPD developers need to take into account the complex adaptive systems framework, understand behaviour change psychology and integrate learning into action through the use of quality improvement strategies
• CPD developers are agents of change in the healthcare system influencing clinical practice and system improvements through the use of innovative strategies in their professional development initiatives

## Notes On Contributors

*Natalie Ong MBBS, FRACP, MClinEd* is a staff specialist developmental paediatrician, at the Children’s Hospital at Westmead, Sydney Children’s Hospitals Network. She is also Clinical Lecturer, Children’s Hospital Westmead Clinical School, University of Sydney and Adjunct Lecturer for the School of Women’s and Children’s Health, University of New South Wales. One of her special interests is in post-graduate clinical education and developing new approaches to health workforce education and training. ORCID: https://orcid.org/0000-0002-0962-443X

*Janet Long* is a Research Fellow at the Australian Institute of Health Innovation, Faculty of Medicine and Health Sciences at Macquarie University. She is a member of the Complexity Science group that uses a complexity lens to approach issues of quality and safety in the delivery of healthcare. Her research interests are around moving research into practice, interdisciplinary relationships and the impact of new technologies on real world practice. ORCID: https://orcid.org/0000-0002-0553-682X

*James N. Woodruff, MD*, Professor Woodruff serves as Dean of Students for the University of Chicago’s Pritzker School of Medicine and is a former Program Director for the Internal Medicine Residency. His areas of interest in medical education are professional development and specifically complex problem solving and adaptive behaviour. He practices general internal medicine in both the inpatient and outpatient settings. ORCID: https://orcid.org/0000-0001-6050-820X
