# Continuing Professional Development through the lens of complexity science: Becoming agents of change in the healthcare system

**DOI:** 10.15694/mep.2020.000186.2

**Published:** 2021-09-27

**Authors:** Natalie Ong, Janet Long, James Woodruff

**Affiliations:** 1 Children's Hospital at Westmead Clinical School; 2 Australian Institute of Health Innovation; 3 Australian Institute of Health Innovation; 4 Pritzker School of Medicine; 5 Pritzker School of Medicine

**Keywords:** continuing professional development, in service training, complex adaptive systems, complexity science, staff education, quality improvement, intellectual disability, developmental disability, children

## Abstract

This article was migrated. The article was marked as recommended.

Healthcare improvement initiatives have not led to expected changes in patient population outcomes over the last decade. This can, in part, be explained by overly reductionist approaches in medical practice and education. This article describes some insights and experiences using a Complex Adaptive Systems approach in the conceptualisation of an in-service training program and reflections on elements that may lead to enhanced practice behaviours and system improvements.

## Introduction

Through the decades, progress in health care quality and performance has been known to be slow. In an article in the BMJ and The Health Foundation Quality Improvement series (2018), Braithwaite stated that only “50-60% of care is delivered using level 1 evidence or clinical guidelines; a third of medicine is waste and the rate of adverse events for the last 25 years have remained static at about 10%” ( Braithwaite, 2018). The paper calls for alternate ways of looking at the healthcare system in order to devise new solutions for healthcare challenges ( Braithwaite, 2018). There are many hypotheses as to why healthcare quality has not improved as expected. Successful problem solving in complex human systems requires an accounting of interactions between human-performance, healthcare delivery process and structure-environmental factors ( Brady *et al*., 2009). Amalberti and colleagues (2011) ascribe the lack of decline in adverse events and waste, despite aggressive patient safety policy and interventions to safety models and tools that are reductionist ( Amalberti *et al*., 2011). These policies, it is argued, fail to account for the varied and often discordant perspectives of human participants within the health organization ( Thomas, 1966). Other contributing factors are social ( Wailling *et al*., 2020), cultural ( Braithwaite *et al*., 2017), and historical ( Classen *et al*., 2011). Woodruff suggests that we cannot solve complex problems using a reductionist paradigm because complexity exists within all layers of the healthcare system and affects the efforts of clinicians and policy makers to improve and reconcile individuals’ healthcare experience, population outcomes and healthcare costs ( Woodruff, 2019). To better account for the complex nature of medicine, such systems should instead be viewed through a “complex adaptive systems framework” (CAS). Such a framework better anticipates the dynamic interactions between the individual’s biopsychosocial systems and complex layers of the health system ( Kuziemsky, 2016). Batalden and Davidoff (2007) propose a model that describes the complex relationship between high quality professional development and quality improvement programming characterized by involvement of patients, families, payers, planners, researchers and educators towards improved patient outcomes. They also propose that ‘change’ is dynamic and needs to be an intrinsic part of the healthcare system ( Batalden and Davidoff, 2007). For this to occur, continuing professional development (CPD) activities should include or work synergistically with continuous quality improvement (QI) initiatives characterized by collaborative teams pursuing iterative problem solving.

## Model Framework

Woodruff proposes a model for applying CAS concepts to medicine. The model outlines four system elements required for self-organising systems or resilient medical organisations tasked with complex problem solving. These include “intrinsic characteristics” that contribute to the mission (expertise, knowledge), an “attractor” (shared values, professionalism), “adaptive capacity” (judgment, responsiveness to the surrounding environment), and “the absence of excessive central control” (professional autonomy, space to respond autonomously to local phenomena). Collectively these elements necessarily result in team oriented continuous quality improvement and endow the system with a capacity to provide ethical, coordinated and personalized care. The absence of any one element, compromises that capacity. Using these four system elements the proposed educational model comprises of the use of Motivational Interview (MI), Flipped Classroom (FC) and Process Mapping (PM) in an iterative format with pre and post session evaluations.

A mix of clinical and management staff participate in the CPD-QI sessions. Through reflective practices, case-based discussions and simulation scenarios as a group, these influence intrinsic motivation (using group MI techniques) as well as increase technical expertise (“intrinsic characteristics”) both working alongside each other. Through the sharing of experiences and peer norming, this promotes a sense of a common purpose (“attractor”) which then leads to an increase in the capacity to collectively adapt (“adaptive capacity”) ( Woodruff 2019). This is followed by process mapping discussions around staff and patient feedback. This fosters openness, ownership and autonomy (“absence of excessive central control”) as staff are supported to speak up about areas that need improvement or attention to skills development.

## Examples illustrating concepts

Two departments had volunteered through their managers to participate in Motivated for Change CPD-QI sessions with the staff of their departments. For the session framework please see Supplementary File 1 ( Ong *et al*., 2020).

Kids Research Institute: Two sessions were conducted in July 2019 and follow up after 7 months with 10 participants (manager, research officers and nursing staff). Four families were interviewed for feedback on their experiences of their participant journey post session.

Participants were given pre-reading prior to the sessions about disability and reasonable adaptations. During the session, an introduction about intellectual and developmental disability (ID/DD), their issues in access and equity in healthcare and the implications for enrolling children with ID/ DD in research were covered. A facilitated discussion followed using a hypothetical case about a teenager with ID, autism and challenging behaviours presenting to hospital and staff were encouraged to discuss ways to elicit the patient’s communication, care needs and reasonable adjustments required for a better experience within their context. Participants also shared their own personal experiences of working with children with ID/DD identifying what was working well and the challenges they faced. They reflected on the need for preparation and planning for reasonable adaptations ranging from the way they communicated and interacted with the child to environmental modifications required for improved patient experiences. A process map was drawn up that identified the patient journey (pink), what was working well (green) and what needed to change (orange) and what were the suggested changes (blue). SMART goals were developed and a follow up session after 7 months was organised. Please see Supplementary File 2.

The following outcomes have been elicited during evaluation:

Changes in procedures and practice

• review of the medication policy in regards to storage, monitoring and delivery
• instituted “team talk” for better handover
• introducing parent phone calls to ask for disability related issues
• developing disability specific templates for the patient folder
• modifying the procedure room with distractor toys
• reducing staff number and noise
• reducing waiting and procedural time
• CNC to develop visuals and communication tools

Changes to staff participant confidence and skills

• Staff felt more comfortable interacting with children with ID/DD
• Staff recognising the need to have a calm and friendly approach and use language appropriate to the level of the child
• Staff reporting higher levels of confidence, competency and changes to practice and attributed it to the sessions

Families experiencing change and improvements in clinical practice

• noticing changes in number of staff involved
• less noisy and overwhelming clinical environment
• better use of distractors and rewards
• more timely way of completing invasive tests
• smoother process of getting premeds (though the effect of premeds were variable)
• use of play therapists for reducing anxiety

Feedback loop

• Staff wanted more training in engaging families of children with disabilities (responding to parent feedback)
• Staff recognised the need for more training in lieu of staff turnover
• Staff self-identified that there was more work that needed to be done in enhancing the clinical space
• Staff idea of having a premeds cabinet was not approved which led to them developing an alternative pathway for children to still receive premeds when needed

The program was also run with the medical imaging department.

Medical Imaging: Six sessions over a period of 9 months (May 2019- Feb 2020) - follow up after 6 months with 31 participants (managers, radiographers, sonographers and nursing staff) (see Supplementary File 2 for more details). Eight families were interviewed.

The following outcomes have been elicited during evaluation:

Changes to staff participant knowledge and skills

• Staff report improvements in pre and post knowledge and skills

Feedback loops

• Staff attended training requesting more sessions for those who did not attend

Local Champions and early adopters

• Staff recruited six more colleagues to assist in the development of resources.
• Staff spoke with their department head about the need for [“just” intervention] and consistently maintained this line of communication for the remainder of the project.

Open and just culture and innovative localised solutions

• Staff spoke with their head of department of the need to review their electronic systems to identify children with developmental disability in order to prepare for their imaging procedure
• Head of Department approached another hospital for permission to use their website of animations and videos as a means to introduce child to different types of imaging procedures to alleviate anxiety and promote cooperation (esp. children with developmental disability).

## Discussion and Conclusion

Using this approach, the author (Ong) found that learners have an increased desire to learn more, take action and propose unexpected projects and QI initiatives. These activities done over time have set up “feedback loops” where efforts that resulted in success encouraged further efforts. This then led to the emergence of more open and “just” cultures, local champions and early adopters, innovative localised solutions and system improvements. These behaviours if sustained could potentially spread to other individuals or departments, inspiring the same approach and to adopt the habit of lifelong learning and contribute to continuous quality improvement and the development of a dynamic learning organisation. Our experience is that through this novel educational intervention - using Motivational Interview, Flipped Classroom and Process Mapping techniques, allows for collaborative discourse through shared experiences, iterative learning and action, shows promise in addressing complex problem solving and professional development in highly complex human organizations.

CPD providers are agents of change for our healthcare system. While their efforts have traditionally focused on enhancing professional competency, they are well positioned to go even further, taking a more explicit role in enhancing patient care and health outcomes. Such efforts will require integration of CPD with core concepts from quality improvement such as systems thinking and complexity science ( Sargeant *et al*., 2018). CPD designed with concepts such as CAS in mind has the potential to further transform our healthcare workforce into flexible and adaptable teams committed to a culture of continuous improvement of healthcare for all.

## Take Home Messages

Progress in health care quality and performance has been slow possibly due to overly reductionist approaches to complex issues that inadequately attend to human factors in medical education and clinical practice.

New ways of thinking about the health care system is required to devise new approaches to improvements in health care.

Complexity exists in all layers of the health care system from the biopsychosocial interactions at the level of the individual to the interactions of complex networks within the health system.

CPD developers need to take into account the complex adaptive systems framework, understand behaviour change psychology and integrate learning into action through the use of quality improvement strategies.

CPD developers are agents of change in the healthcare system influencing clinical practice and system improvements through the use of innovative strategies in their professional development initiatives.

## Notes On Contributors

Natalie Ong MBBS, FRACP, MClinEd is a staff specialist developmental paediatrician, at the Children’s Hospital at Westmead, Sydney Children’s Hospitals Network. She is also Clinical Lecturer, Children’s Hospital Westmead Clinical School, University of Sydney and Adjunct Lecturer for the School of Women and Children’s Health, University of New South Wales. One of her special interests is in post-graduate clinical education and developing new approaches to health workforce education and training. ORCiD: https://orcid.org/0000-0002-0962-443X

Janet Long is a Research Fellow at the Australian Institute of Health Innovation, Faculty of Medicine and Health Sciences at Macquarie University. She is a member of the Complexity Science group that uses a complexity lens to approach issues of quality and safety in the delivery of healthcare. Her research interests are around moving research into practice, interdisciplinary relationships and the impact of new technologies on real world practice. ORCiD: https://orcid.org/0000-0002-0553-682X

James N. Woodruff, MD, Professor Woodruff serves as Dean of Students for the University of Chicago Pritzker School of Medicine and is a former Program Director for the Internal Medicine Residency. His areas of interest in medical education are professional development and specifically complex problem solving and adaptive behaviour. He practices general internal medicine in both the inpatient and outpatient settings. ORCiD: https://orcid.org/0000-0001-6050-820X
