# Supplementary material for: Continuing Professional Development through the lens of complexity science: Becoming agents of change in the healthcare system
Source: MedEdPublish (2016). 2021 Sep 27;9:186. Originally published 2020 Aug 31. [Version 2] doi: 10.15694/mep.2020.000186.2 (PMC10699371; doi:10.15694/mep.2020.000186.2)
Supplement: Peer review history

# Reviewer report: Reviewer response for version 2

**DOI:** 10.21956/mep.20278.r31481

**Published:** 2021-10-06

**Authors:** Trevor Gibbs

**Affiliations:** 1 AMEE

This review has been migrated. The reviewer awarded 3 stars out of 5 Having read the first and second version of this paper, I am still at a loss to see the final outcome of this work. Much of the evaluation of the activity was given over to self-reflection, when I think that a higher level of evaluation could have been performed over time. I do not feel that the authors have answered all of the questions / suggestions asked by one of the first reviewers of this paper.

Reviewer Expertise:

NA

No decision status is available

# Reviewer report: Reviewer response for version 2

**DOI:** 10.21956/mep.20278.r31480

**Published:** 2021-10-03

**Authors:** Ken Masters

**Affiliations:** 1 Sultan Qaboos University

This review has been migrated. The reviewer awarded 4 stars out of 5 The authors have addressed my major concern of Version 1 by supplying practical examples, both in the text and in the supplementary files. The paper is now much stronger, and is a valuable opinion piece contribution to the field.

Reviewer Expertise:

NA

No decision status is available

# Reviewer report: Reviewer response for version 1

**DOI:** 10.21956/mep.19139.r27657

**Published:** 2020-11-28

**Authors:** Ken Masters

**Affiliations:** 1 Sultan Qaboos University

This review has been migrated. The reviewer awarded 3 stars out of 5 An interesting opinion piece on the application of a Complex Adaptive Systems Framework to CPD.While the piece is interesting, it will leave many readers asking the question “How is this to be done?” I think that the paper would be greatly strengthened if the authors could supply a practical example of a course utilising the theory. Because it is an opinion piece, it would not be necessary to perform evaluations, but would offer a little more practical insight to its application.Other issues have been raised by a previous reviewer, so there is no need for me to raise them here.I look forward to Version 2 of the paper in which these issues are addressed.

Reviewer Expertise:

NA

No decision status is available

# Reviewer report: Reviewer response for version 1

**DOI:** 10.21956/mep.19139.r27658

**Published:** 2020-09-05

**Authors:** Megan Anakin

**Affiliations:** 1 University of Otago

This review has been migrated. The reviewer awarded 2 stars out of 5 I read your personal view/opinion piece with interest because one of my roles at my university is as a staff developer. I am also interested in theoretical tools that help us study the complexity when providing healthcare. Overall, I felt that this article is quite brief and might benefit with further description and examples from practice so the reader can better appreciate how the ideas presented can be applied to their contexts and so the article better achieves the aims set out in the abstract by providing more detailed insights, descriptions of experiences, and specifics about the reflections of the authors.The authors may wish to consider taking a complexity approach to presenting their argument as to “why healthcare quality has not improved as expected”. They focus on one hypothesis to suggest that the slow pace of healthcare improvement may be due to over-reliance on reductionist approaches. The authors may wish to present references from the staff development literature that suggest healthcare improvement may also involve social, cultural, and historical factors. This expanded introduction would help demonstrate that there is complexity involved in understanding why healthcare quality has not improved as expected, in addition to their argument about using complexity science to enhance CPD efforts.To help the reader appreciate how CPD and QI activities might be aligned, the authors may wish to present a practical example in the second paragraph of the article.To help the reader understand the context of the example from personal experience, please identify the author represented by “my” and “I” in the first sentence of the fourth paragraph. Please also consider providing a description the “process mapping discussion about staff and patient feedback’ so the reader can better understand how the system elements were applied and examples of the areas identified by staff “that need improvement or attention to skills development”. Please provide evidence to support the statement, “Using this approach, I have found that learners have an increased desire to learn more, take action and propose unexpected projects and QI initiatives”. Please provide an example to illustrate how “These activities done over time have set up “feedback loops” where efforts that resulted in success encouraged further efforts.” so that there is evidence presented to support how “This then led to the emergence of more open and “just” cultures, local champions and early adopters, innovative localised solutions and system improvements.”Please consider revising the take home messages to make them more tentative and better reflect that this is a personal view/opinion piece article. For example, please revise the first take home message because there is no evidence presented in the article to support this claim and consider making this statement tentative so it corresponds to your statement about it being a hypothesis in the first paragraph of the article. I would be very happy to read and review a revised version of this article.

Reviewer Expertise:

NA

No decision status is available
